# Impact of Lavender Flower Powder as a Flavoring Ingredient on Volatile Composition and Quality Characteristics of Gouda-Type Cheese during Ripening

**DOI:** 10.3390/foods12081703

**Published:** 2023-04-19

**Authors:** Cristina Anamaria Semeniuc, Mara Mandrioli, Matilde Tura, Beatrice Sabrina Socaci, Maria-Ioana Socaciu, Melinda Fogarasi, Delia Michiu, Anamaria Mirela Jimborean, Vlad Mureşan, Simona Raluca Ionescu, Mihaela Ancuţa Rotar, Tullia Gallina Toschi

**Affiliations:** 1 Faculty of Food Science and Technology, University of Agricultural Sciences and Veterinary Medicine of Cluj-Napoca, 3-5 Calea Mănăştur, 400372 Cluj-Napoca, Romania; cristina.semeniuc@usamvcluj.ro (C.A.S.); socaciubeatricesabrina@gmail.com (B.S.S.); maria-ioana.socaciu@usamvcluj.ro (M.-I.S.); melinda.fogarasi@usamvcluj.ro (M.F.); delia.michiu@usamvcluj.ro (D.M.); mirela.jimborean@usamvcluj.ro (A.M.J.); vlad.muresan@usamvcluj.ro (V.M.); rallucab@yahoo.com (S.R.I.); anca.rotar@usamvcluj.ro (M.A.R.); 2 Centre for Technology Transfer-BioTech, 64 Calea Florești, 400509 Cluj-Napoca, Romania; 3 Department of Agricultural and Food Sciences, Alma Mater Studiorum-Università di Bologna, Viale Giuseppe Fanin 40, 40127 Bologna, Italy; mara.mandrioli@unibo.it (M.M.); tullia.gallinatoschi@unibo.it (T.G.T.)

**Keywords:** lavender-flavored Gouda-type cheese, physicochemical properties, textural properties, microbiological properties, volatile compounds, sensory properties

## Abstract

This study aimed to formulate a Gouda-type cheese from cow’s milk, flavored with lavender flower powder (0.5 g/L matured milk), ripened for 30 days at 14 °C and 85% relative humidity. Physicochemical, microbiological, and textural characteristics, as well as the volatile composition of the control (CC—cheese without lavender) and lavender cheese (LC), were assessed at 10-day intervals of ripening. Consumers’ perception, acceptance, and purchase intention were only evaluated for ripened cheeses. Moisture and carbohydrate contents, the pH, cohesiveness, indexes of springiness and chewiness decreased during ripening in both CC and LC; however, protein, ash, and sodium chloride contents, titratable acidity, hardness, lactobacilli, streptococci, and volatiles increased. Fat and fat in dry matter contents, respectively, the energy value did not vary with ripening time in LC and increased in CC; gumminess decreased in CC and did not change in LC. Lavender flower powder significantly affected the cheese’s microbiological and sensory characteristics and volatile composition but did not considerably impact physicochemical and textural ones. Populations of lactobacilli and streptococci were substantially higher in LC compared to CC. The volatile profile of LC was dominated by terpene and terpenoids, and that of CC by haloalkanes. Sensory scores were slightly lower for LC than CC, even if it did not considerably affect consumers’ acceptance and purchase intention.

## 1. Introduction

Gouda is a ripened firm/semi-hard cheese with a body color ranging from near-white or ivory to light yellow or yellow and a firm texture. Its interior has a few to plenty of gas holes uniformly distributed. This type of cheese has a smooth and dry rind, with few openings and splits accepted [1]. It is ordinarily made from pasteurized cow’s milk and acidified with a mesophilic starter culture containing miscellaneous lactic acid bacteria [2]. Gouda cheese generally has an inside diameter of approximately 25.4 cm and a thickness of 16.5 cm. The percentage of water varies from 41.25 to 45.43%, with an average of 43.5% [3].

Furthermore, since several brands of Gouda-type cheese are commercially available, the sensory properties, mainly the cheese flavor, are the key factors affecting consumers’ acceptance and are decisive for purchase intention [4,5]. Thus, dairy manufacturers have started producing differentiated cheeses by incorporating some atypical ingredients, such as lavender [6], cumin [7], red chili pepper [8], fenugreek [9], mustard [10], and garlic [10]. However, despite the potential appeal of these cheeses to consumers, there needs to be more knowledge regarding the effects of adding these flavoring ingredients on the development of texture and flavor in ripened cheese [6].

It is well known that microorganisms, especially those in the starter culture, play an essential role in cheese making; the enzymes produced by them break down cheese constituents such as lipids, proteins, and, to a lesser extent, carbohydrates, improving the product texture and flavor during ripening [11]. The starter culture used in Gouda-type cheese manufacturing includes *Lactococcus*, *Leuconostoc*, *Lactobacillus*, and *Streptococcus* strains [12]. Members of the *Leuconostoc* genus and lactococcal variant *Lactococcus lactis* subsp. *lactis* biovar. *diacetylactis* ferment milk citrate into various compounds, the most significant being carbon dioxide (necessary for eye formation in Gouda cheese) and the diacetyl and acetic acid flavors [13].

The antimicrobial properties of some spices/herbs used as ingredients are widely known, and, thus, their possible effects on starter organisms in cheese. On the other hand, the microbiome of these flavoring ingredients also affects the starter culture during ripening, and hence the development of the cheese’s texture and flavor [11]. However, to our knowledge, work has yet to be published regarding the impact of lavender flower powder on the Gouda-type cheese’s ripening process and implicitly on its properties. Therefore, this study proposes a manufacturing process for a Gouda-type cheese flavored by adding lavender flower powder into matured milk to evaluate the effect of lavender flower powder and the ripening time on the cheese’s volatile compounds and the physicochemical, textural, and microbiological characteristics.

## 2. Materials and Methods

Raw milk (3.8% fat, 3.4% protein, 4.5% lactose, 87.5% moisture, and a pH of 6.5) from Bălțată Românească cattle was used to manufacture the Gouda-type cheeses (LC—with lavender flower powder and CC—without the flavoring ingredient). Cow’s milk was received (as a donation) from a milk farm (P.F.A. Socaci L. Maria) in Chirileu, Mureș County, Romania, with a herd of 30 cows (1–6 lactation cycles). Cows were kept in free stabulation during the daytime to graze on green pastures. As additional roughage, they were fed with a mixture of alfalfa, hay, orchard grass, and silage or a blend of corn, wheat, sunflower, and barley flour.

Lavender (dried bunches of *Lavandula angustifolia* Mill.) was purchased from a lavender farm (Lavanda Lola) in Bonțida, Cluj County, Romania. The flavoring ingredient, lavender flower powder, was prepared by grinding lavender flowers (manually detached from stems and separated from impurities) to a fine powder using a mortar grinder (RM 200; Retsch GmbH, Haan, Germany); the powder thus obtained was then sealed into a glass jar with a lid and kept in a cool, dry place until use.

### 2.1. Manufacturing of Gouda-Type Cheese

**Figure 1:**
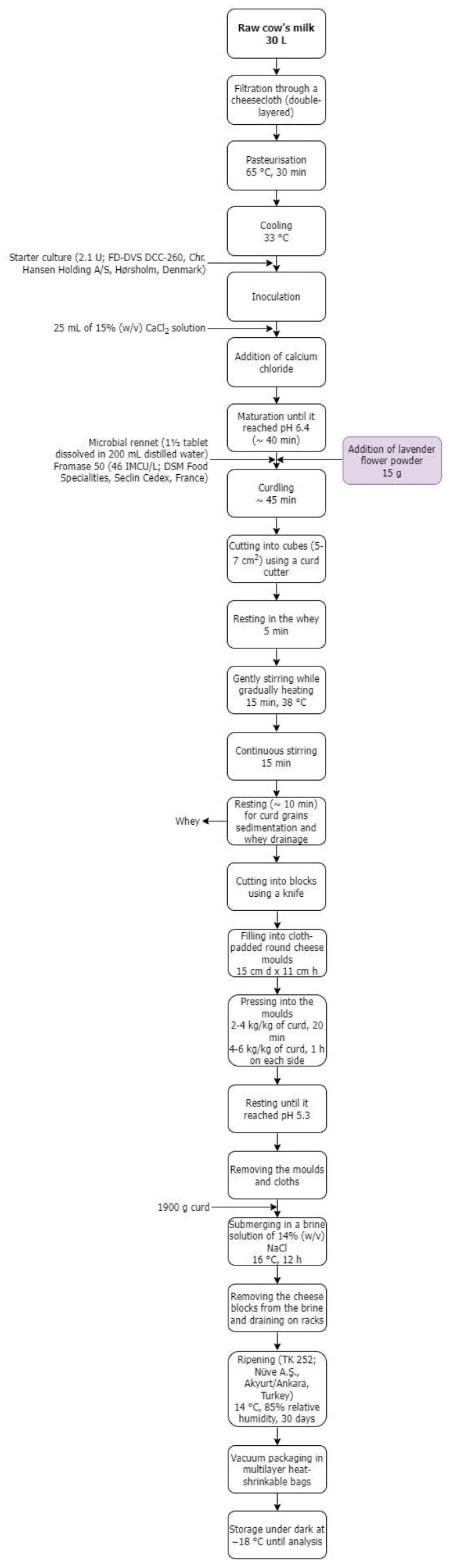
Flowchart of lavender Gouda-type cheese manufacturing process.

**Figure 2:**
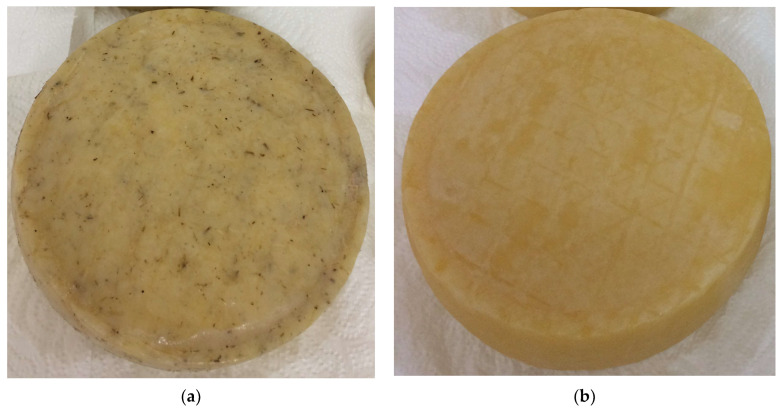
Gouda-type cheeses: (**a**) LC—lavender cheese; (**b**) CC—control cheese.

Gouda-type cheese was made as described in our previous paper [6]. An average quantity of 3.85 kg CC and 3.95 kg LC was obtained from processing 30 L cow’s milk. The flowchart in Figure 1 shows the manufacturing process steps for LC (see Figure 2a); CC (see Figure 2b) was manufactured similarly but without lavender flower powder. Both treated (LC) and untreated (CC) cheeses were produced in two batches. The amount of lavender flower powder (15 g) added into matured milk (30 L) to flavor the cheese was selected from a series of tested concentrations (30, 25, 20, and 15 g lavender powder per 30 L of milk) based on a sensory evaluation of cheese (performed using an internal, trained panel of 6 assessors).

All analyses were performed both on the lavender (LC) and control cheese (CC) at ten (T1), twenty (T2), and thirty days (T3) of ripening, as mentioned in Section 2.2, Section 2.3, Section 2.4, Section 2.5 and Section 2.6. In addition, consumers’ perception, acceptance, and purchase intention were also determined for ripened LC and CC, as described in Section 2.7.

### 2.2. Proximate Composition Analysis of Cheese and Calculation of Total Carbohydrate Content, Fat in Dry Matter Content, and Energy Value

Moisture content was determined by drying the sample to a constant weight in an electric oven (Digitheat; J.P. Selecta S.A., Barcelona, Spain), following instructions provided by ISO 5534:2004|IDF 4:2004 [14].

Determination of the nitrogen content and calculation of crude protein was carried out as described in ISO 8968-1:2014|IDF 20-1:2014 [15] using the Kjeldahl method. It involved acid digestion of the sample (DK6 Heating Digester; VELP Scientifica SRL, Usmate Velate, Italy), followed by alkalization and steam distillation of the acid digest (UDK 129 Distillation Unit; VELP Scientifica S.R.L., Usmate Velate, Italy), and finally by quantification of the trapped ammonia through titration. The protein content in cheese was estimated by multiplying the total nitrogen by 6.25, a nitrogen-to-protein conversion factor.

Fat content was determined directly using the Van Gulik method from ISO 3433:2008|IDF 222:2008 [16], while fat in dry matter (FDM) content was calculated by the following Formula (1):

(1) $$FDM\;\left(\%\right)=\frac{F}{DM}\times 100$$

where F is the fat content (%) of the cheese, and DM is the dry matter content (%) of the cheese (computed by subtracting moisture content (%) from 100).

Ash content was determined by incineration of the sample in a muffle furnace (L3/11/B170; Nabertherm GmbH, Bremen, Germany), as detailed by Nagy et al. [17]. First, approximately 1.0 g of grated cheese was weighed to the nearest 1 mg (ABJ-220-4NM; Kern & Sohn GmbH, Balingen, Germany) into a porcelain crucible, and it was then heated at 600 °C for 12 h in the muffle furnace, cooled in a desiccator, and weighed again. Finally, the percentage of ash content was calculated with the following Formula (2):

(2) $$Ash\;\left(\%\right)=\frac{w_{a}}{w_{s}}\times 100$$

where $w_{a}$ is the weight (g) of ash, and $w_{s}$ is the weight (g) of the sample.

All samples were analyzed in triplicate, and results were expressed in percentages.

Total carbohydrate content (%) was calculated from Formula (3) used by Nagy et al. [17]:

(3) $$Total\;carbohydrate\;\left(\%\right)=100-\left(\%\;\mathrm{moisture}+\%\;\mathrm{protein}+\%\;\mathrm{fat}+\%\;\mathrm{ash}\right)$$

The energy value of cheese was calculated according to Nagy et al. [17] using the following Formula (4):

(4) $$Energy\;value\;\left(\mathrm{kcal}/100\;g\right)=4\times \left(g\;protein+g\;carbohydrate\right)+9\times \left(g\;fat\right)$$

### 2.3. Determination of Sodium Chloride Content, pH, and Titratable Acidity of the Cheese

Sodium chloride content, expressed as a percentage, was determined following the potentiometric titration method specified in ISO 5943:2006|IDF 88:2006 [18]. The pH measurement of cheese was performed with a portable pH meter (HI 99161; Hanna Instruments, Limena, Italy). Titratable acidity, expressed in Thörner degrees (°T), was determined using the titrimetric method from STAS 6353-85 [19], a Romanian standard. All samples were analyzed in triplicate.

### 2.4. Texture Profile Analysis of Cheese

This analysis included measurement of the hardness (N), cohesiveness, springiness index, gumminess (N), and chewiness index (N) of cheese sample using a texture analyzer (CT3; Brookfield Engineering Laboratories Inc., Middleboro, MA, USA), according to the method described by Ong et al. [20]. Samples, in 1.5 cm cubes, were taken from the central part of the cheese using a knife and kept at 20 °C for 1 h in a closed container (to prevent moisture loss) until analysis. The test consisted of sample deformation to 50% of its height (7.5 mm), at a speed of 2 mm/s, with a TA25/1000 cylindrical probe attached to a 10 kg compression cell. Measurements were carried out on each cheese batch in triplicate (six in total for each treatment) using the TexturePro CT software.

### 2.5. Microbiological Analysis of Cheese

A portion of 5.0 g grated cheese was aseptically weighed into a sterile stomacher bag and homogenized in 45 mL of 0.85% (*w*/*v*) sodium chloride solution (27810.295P; VWR Chemicals, Leuven, Belgium) for 1 min using a laboratory blender (MiniMix 100 P CC; Interscience, Saint-Nom-la-Bretèche, France), as described by Socaciu et al. [21]. Seven ten-fold serial dilutions (10^−2^, 10^−3^, 10^−4^, 10^−5^, 10^−6^, 10^−7^, and 10^−8^) were prepared from this stock solution (10^−1^).

Lactobacilli and streptococci counts were determined in cheese according to the method described by the ISO 7889:2003|IDF 117:2003 standard [22] using MRS broth (110611; Merck KGaA, Darmstadt, Germany) and M17 agar (CM0785; Oxoid Ltd., Basingstoke, England), respectively. Enumeration of lactobacilli and streptococci colonies was performed after incubating inoculated plates at 37 °C for 48 h under anaerobic conditions. All samples were analyzed in triplicate, and results were reported as log cfu/g. In addition, the total lactic acid bacteria count (log cfu/g) was calculated as the sum of the lactobacilli and streptococci counts.

### 2.6. Analysis of Volatile Compounds in Cheese

This was performed following the method described by Cozzolino et al. [23], with minor modifications. Into a 20-mL SPME crimp neck vial (22.5 × 75.5 mm; VWR International s.r.l., Milano, Italy), approximately 3.5 g of grated cheese was weighed to the nearest 0.1 mg using an analytical balance (E42; Gibertini Elettronica S.R.L., Milano, Italy); 3 mL aqueous solution of hydrochloric acid (0.03% 6 M HCl; Merk KGaA, Darmstadt, Germany) was added to cheese sample and mixed, followed by 5 µL methanolic solution of methyl nonanoate (1175 μg/mL; Sigma-Aldrich Chemie GmbH, Steinheim, Switzerland), used as an internal standard. First, the vial’s content was stirred and then kept for 30 min at 40 °C in the autosampler thermostat (HT2850T autosampler; HTA S.r.l., Brescia, Italy) to reach equilibrium. Next, an SPME fiber (50/30 µm DVB/CAR/PDMS; Supelco Inc., Bellefonte, PA, USA) was inserted into the vial’s septum and exposed to the sample headspace at 40 °C for 30 min to adsorb the volatile compounds. Before its first use, the fiber was conditioned at 270 °C for 60 min, according to the manufacturer’s recommendations. After sampling, it was retracted and automatically injected into the gas chromatograph injection port of a GCMS-QP2010 Plus instrument (Shimadzu Corp., Kyoto, Japan) equipped with an Rtx-Wax capillary column (Crossbond^®^ Carbowax^®^ polyethylene glycol; 30 mL × 0.25 mm ID × 0.25 μm film thickness; Restek Corp., Bellefonte, PA, USA), where volatile compounds were desorbed for 10 min in spitless mode. Helium was the carrier gas at 1 mL/min constant flow. The injector temperature was set to 250 °C, and the oven one was programmed initially at 40 °C and held at this temperature for 2 min. Subsequently, it was increased by 5 °C/min up to 65 °C and kept at 65 °C for 2 min, and then by 10 °C/min up to 240 °C and held at 240 °C for 9 min. Interface and ion source temperatures were set to 210 and 230 °C, respectively, and the filament voltage to 70 eV (electronic impact). Chromatographic analysis was carried out in triplicate for each cheese sample. Volatile compounds were identified by comparing their recorded mass spectra with those found in the NIST27 and NIST147 libraries. The relative content of each volatile compound was calculated as the ratio of its total ion current (TIC) area to the TIC area of the internal standard using the following Formula (5):

(5) $$Conc\;A\;\left(µg/\mathrm{kg}\;cheese\right)=\left(\frac{Peak\;area_{A}}{Peak\;area_{IS}}\times \frac{a_{IS}}{w_{c}}\right)\times 1000$$

where Conc A is the analyte concentration, $Peak\;area_{A}$ is the analyte peak area, $Peak\;area_{IS}$ is the internal standard peak area, $a_{IS}$ is the amount of internal standard added to the sample (µg), and $w_{c}$ is the cheese weight (g).

### 2.7. Sensory Analysis of Cheese and Determination of Consumers’ Acceptance and Purchase Intention

Cheese samples were assessed for appearance and color, consistency and texture, odor and taste, aftertaste, and overall liking using a nine-point hedonic scale (1—dislike extremely; 2—dislike very much; 3—dislike moderately; 4—dislike slightly; 5—neither like nor dislike; 6—like slightly; 7—like moderately; 8—like very much; 9—like extremely). They were coded with 3-digit random numbers and presented to panelists (50 women and 30 men aged 20–43 years) on white ceramic plates. The sensory attributes of CC and LC were rated at 20 °C (air conditioning) under white light. Panelists were asked not to eat, drink, or smoke for at least 1 h before the evaluation session (conducted in individual booths).

Consumers’ purchase intention was rated on a 5-point scale (1—definitely will buy; 2—probably will buy; 3—might or might not buy; 4—probably will not buy; 5—definitely will not buy) [24]. The acceptance rate (AR) of each cheese type was calculated as described previously by dos Reis Santos et al. [25] using the following Formula (6):

(6) $$AR\;\left(\%\right)=\frac{X}{N}\times 100$$

where X is the mean sensory score of the cheese, and N is the maximum sensory score given by panelists to the cheese.

### 2.8. Statistical Analysis

The Minitab statistical software (version 19.1.1; LEAD Technologies, Inc., Charlotte, NC, USA) was used for data analysis. The effects of the lavender flower powder and ripening time on the Gouda-type cheese’s characteristics and volatile compounds were analyzed by one-way ANOVA with a post-hoc Tukey’s test at a 95% confidence level (*p* < 0.05). In addition, hierarchical cluster analysis (HCA) was performed using the MetaboAnalyst software (version 5.0; Xia Lab at McGill University, Montreal, QC, Canada).

## 3. Results and Discussion

### 3.1. Nutritional Properties of Gouda-Type Cheese

Changes in the proximate composition, sodium chloride content, pH, titratable acidity, and energy value of the control (CC) and lavender Gouda-type cheese (LC) at 10-day intervals during 30 days of ripening are shown in Table 1. Moisture content significantly decreased with ripening time, from 36.84 to 34.97% in CC and from 37.87 to 35.48% in LC, causing an increase in protein (from 21.13 to 23.17% in CC; from 21.55 to 24.90% in LC), ash (from 4.28 to 5.45% in CC; from 4.25 to 5.06% in LC), and sodium chloride contents (from 4.28 to 5.45% in CC; from 4.25 to 5.06% in LC). On the other hand, the fat content of the Gouda-type cheese increased in CC (from 28.25 to 30.75%) while this matured but did not vary significantly in LC (from 29.25 to 29.75%); the same trends were noticed for the fat in dry matter content and energy value of CC and LC, respectively (see Table 1). Regarding carbohydrates, their content significantly declined in both CC and LC during ripening, as lactic acid bacteria consumed them, causing a fall in pH (from 4.87 to 4.60 in CC; from 4.99 to 4.65 in LC) and an increase in titratable acidity (from 85.2 to 95.0 °T in CC; from 79.8 to 92.0 °T in LC). It is well known that lactic acid bacteria use carbohydrates as a primary carbon source [26], lactic acid being the major end-product of milk lactose fermentation [27]. These results are corroborated by microbiological findings showing the multiplication of lactic acid bacteria in CC and LC with ripening. Furthermore, at the ripening period’s end, protein and sodium chloride contents in LC were significantly higher than in CC, while the ash content and titratable acidity were lower. Nevertheless, both CC and LC met the quality requirements in the Codex standard for Gouda, namely CXS 266-1966 [1].

### 3.2. Textural Properties of Gouda-Type Cheese

Instrumental measurements of texture attributes in CC and LC are presented in Table 2. Consistent with the findings of Kanawjia et al. [28] on Gouda cheese, the hardness, springiness index, gumminess, and chewiness decreased in CC and LC during ripening, except for gumminess in LC, which did not change significantly. However, no significant differences between the texture attribute values of CC and LC were found at the final stage of ripening.

Hardness indicates the maximum force required to compress cheese between the molar teeth. In CC, it significantly increased from a value of 35.43 N on day 10 of ripening to 53.07 N on day 30, and in LC, it raised from 24.72 to 41.51 N, most likely due to a loss of moisture.

Cohesiveness, also known as consistency, shows the strength of the internal bonds making up a cheese’s body. Surprisingly, and contrary to the findings of Kanawjia et al. [28], we noticed a downward trend with ripening time for both CC (from 0.46 to 0.24) and LC (from 0.44 to 0.24). Nevertheless, Ivanov et al. [29] reported changes in the cohesiveness of Kashkaval cheese during ripening, like those we observed, being attributed to proteolysis.

The springiness index is a texture attribute that shows the viscoelastic properties of cheese and ranges from 0 (completely viscous material) to 1 (completely elastic material). As can be seen in Table 2 below, it significantly decreased during ripening, from 0.73 to 0.54 in CC and from 0.82 to 0.68 in LC. Our results are in accordance with those reported by Zheng et al. [30] and reveal that the low moisture content in Gouda-type cheese is associated with high firmness but low springiness and cohesiveness.

Gumminess is the energy required to disintegrate cheese into a state ready for swallowing. Its level significantly decreased in CC as it ripened (from 16.09 to 8.94 N), as in the study of Pinto et al. [31], while in LC (from 10.72 to 10.17 N), it did not vary considerably.

Chewiness indicates the energy required to chew cheese to a state whereby it is ready for swallowing. The chewiness index is estimated as hardness × cohesiveness × springiness index. It decreased with ripening time, from 13.35 N in CC and 8.57 N in LC to 8.57 and 6.96 N, respectively. In another study, Pinto et al. [31] also noticed a decreasing change in cheese chewiness during ripening.

### 3.3. Microbiological Properties of Gouda-Type Cheese

The starter culture used for Gouda-type cheese-making contains a mixture of thermophilic and mesophilic bacteria such as *Lactococcus lactis* ssp. *cremoris*, *Lactococcus lactis* subsp. *lactis*, *Lactococcus lactis* subsp. *lactis* biovar. *diacetylactis*, *Lactobacillus helveticus*, *Lactobacillus paracasei*, *Leuconostoc* species, and *Streptococcus thermophilus*. Therefore, the effect of lavender flower powder on lactic acid bacteria growth in Gouda-type cheese was evaluated by determining the lactobacilli and streptococci count, also, the total lactic acid bacteria count (see Table 3).

The antibacterial effect of lavender against *Escherichia coli*, responsible for early cheese blowing, and *Clostridium tyrobutiricum*, responsible for late cheese blowing, was reported by Librán et al. [32] in a previous study. Therefore, we assumed that lavender flower powder, used as a flavoring ingredient in our Gouda-type cheese, could inhibit the growth of starter culture microorganisms during ripening. Nevertheless, contrary to our expectation, it stimulated the development of lactic acid bacteria since the counts of lactobacilli, streptococci, and total lactic acid bacteria were significantly higher in LC at all ripening times.

However, in line with the study of Öztürk et al. [33], the flavoring ingredient reported herein caused a significant increase in the lactobacilli and streptococci count in CC and LC with ripening time (in CC, from 9.5 to 9.8 log cfu/g, and in LC, from 9.7 to 9.9 log cfu/g for lactobacilli count; in CC, from 8.7 to 9.3 log cfu/g, and in LC, from 9.0 to 9.6 log cfu/g for streptococci count; in CC, from 18.2 to 19.1 log cfu/g, and in LC, from 18.7 to 19.5 log cfu/g for total lactic acid bacteria count). These results also explain the below-discussed accumulation of volatile compounds during cheese ripening in both CC and LC.

### 3.4. Volatile Compounds in Gouda-Type Cheese

**Figure 3:**
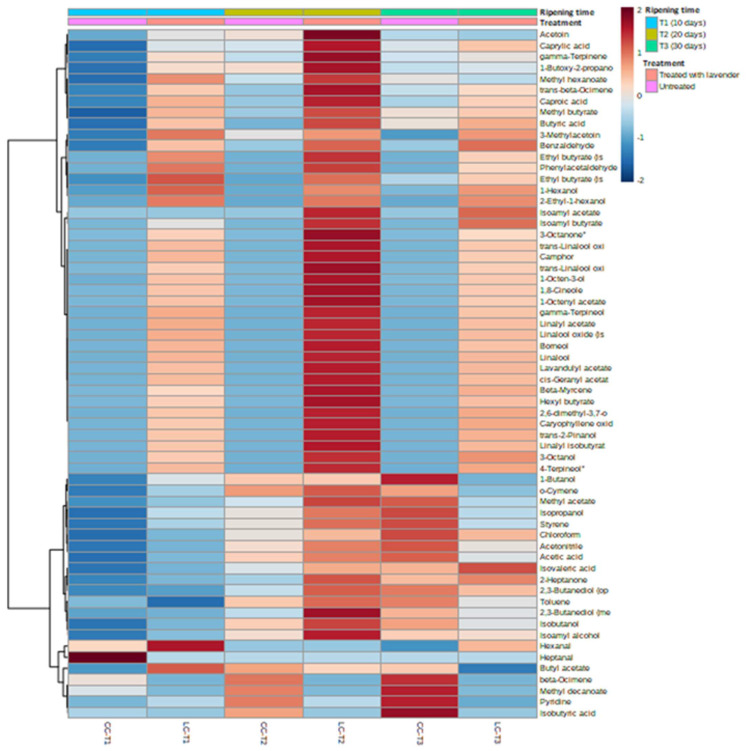
HCA heatmap of Gouda-type cheese based on volatile constituents.

Results of headspace-SPME-GC/MS analysis for quantifying volatile compounds in the Gouda-type cheeses are shown in Table 4. Thirty-seven volatile compounds were detected in CC at T1, classified under six different identified compounds: haloalkane (32.0%—one compound) was the most dominant group, followed by alcohols (23.2%—nine compounds), carboxylic acids (15.4%—six compounds), cyanoalkanes (8.3%—one compound), esters (8.1%—six compounds), and ketones (5.9%—three compounds). Moreover, five compounds were classified within the chemical class of terpenes and terpenoids (3.3%), three in aldehydes (2.2%), two in aromatic hydrocarbons (1.2%), and one in pyridines (0.4%). To better visualize the similarities and differences between cheese samples at different ripening stages regarding the volatile composition, hierarchical cluster analysis (HCA) was run (see Figure 3).

Control cheese. Chloroform (32.0%; No. **5** in Table 4) was the most dominant compound present in CC at T1, followed by acetonitrile (8.3%; No. **4**), isopropanol (6.6%; No. **2**), 1-butanol (6.0%; No. **12**), isoamyl alcohol (5.8%; No. **19**), caproic acid (5.6%; No. **59**), acetoin (5.6%; No. **28**), and methyl hexanoate (4.2%; No. **17**); of these, chloroform, toluene, acetonitrile, and isopropanol were found in the Gouda-type cheese for the first time. Previous findings have revealed the presence of chloroform in semi-hard goat cheese (Kınık et al. [34]), chloroform and toluene in some varieties of Turkish cheese (Hayaloglu and Karabulut [35]), and acetonitrile and isopropanol in Manchego cheese (Gómez-Ruiz et al. [36]). It is well known that chloroform and acetonitrile derive from the breakdown of milk carotene [35,36]. Chloroform may also derive from the chlorine-containing cleaning products used for cheese-processing equipment disinfection [37]. Alcohols (23.2%), the second most dominant class in CC at T1, would be the consequence of the abovementioned compounds’ abundance, together with the presence of optical (0.9%) and meso isomers (2.4%) of 2,3-butanediol. Meanwhile, 1-butanol, isoamyl alcohol, and 2,3-butanediol were also discovered in Gouda-type cheese by Van Leuven et al. [38] and Van Hoorde et al. [39]. Some authors reported that they arise from butanal (resulting from fatty acid or amino acid metabolism), leucine, and acetoin, respectively [36,40,41]. Carboxylic acids (15.4%), the third class in abundance in CC at T1, were represented by acetic acid (2.8%), butyric acid (2.4%), and caproic acid (10.2%), all previously reported in Gouda-type cheese by other studies [38,39,42,43,44,45,46]. They can originate from milk fat lipolysis or lactose and lactic acid fermentation [35]. Cyanoalkanes (8.3%), esters (8.1%), ketones (5.9%), terpene and terpenoids (3.3%), aldehydes (2.2%), aromatic hydrocarbons (1.2%), and pyridines (0.4%) were the subsequent classes of volatile compounds grouped in CC at T1. Acetoin, listed among the significant volatile constituents in CC at T1, is a volatile compound from the ketone class. It was identified as a derivative of milk citrates [36] and, therefore, detected in Gouda-type cheese by other researchers [38,45,46]. Methyl hexanoate (4.2%), from the ester class, another notable volatile compound in CC at T1, was also reported in some commercial Gouda cheeses [2]. Its occurrence is probably related to the esterase activity of lactic acid bacteria [36]. As for aldehydes in cheese, they are produced by the catabolism of fatty acids or amino acids via decarboxylation or deamination [35,47]; only benzaldehyde (1.5%), hexanal (0.2%), and heptanal (0.5%) were detected in CC at T1, but it seems that they are commonly present in Gouda-type cheeses [38,39,45]. Both hexanal and heptanal disappeared during ripening; heptanal was no longer detected in CC on the 20th day of maturation and hexanal on the 30th—hence the lower number of volatile compounds found in CC at T2 (thirty-six) and CC at T3 (thirty-five). *o*-Cymene (1.7%), *β*-ocimene, trans-*β*-ocimene (0.6%), *ɣ*-terpinene (0.2%), and linalool (0.3%), volatile compounds of the terpene and terpenoid class, were also present in CC at T1; since this is the first time that they have been detected in a Gouda-type cheese, they most likely arise from animal feed, considering that the cows had pasture access in the afternoon and evening.

Lavender cheese. The volatile profile of LC at T1 (Table 4), however, was dominated by terpenes and terpenoids (Te&Ts; 42.3%), followed by alcohols (Alc; 15.5%), haloalkanes (HoAlka; 13.3%), carboxylic acids (CA; 11.6%), esters (Es; 7.8%), ketones (Ket; 4%), cyanoalkanes (CyAlka; 3.5%), aldehydes (Ald; 1.6%), aromatic hydrocarbons (AH; 0.2%), and pyridines (Pyr; 0.1%). Sixty-two volatile compounds were detected in this sample, including 12 alcohols, 3 aldehydes, 1 aromatic hydrocarbon, 5 carboxylic acids, 1 cyanoalkane, 10 esters, 1 haloalkane, 4 ketones, 1 pyridine, and 19 compounds from the terpene and terpenoid class, with three more alcohols than in CC at T1 (3-octanol; 2-ethyl-1-hexanol; 2,6-dimethyl-3,7-octadien-2,6-diol), 1 aldehyde (phenylacetaldehyde), 5 esters (an isomer of ethyl butyrate; isoamyl acetate; isoamyl butyrate; 1-octenyl acetate; hexyl butyrate), 1 ketone (3-octanone), and 15 compounds of the terpene and terpenoid class (*β*-myrcene; 1,8-cineole; 3 isomers of linalool oxide; trans-2-pinanol; linalyl acetate; camphor; linalyl isobutyrate; 4-terpineol; lavandulyl acetate; *α*-terpineol; borneol; cis-geranyl acetate; caryophyllene oxide). Moreover, isobutyric acid (CA) was identified along with linalyl isobutyrate (Te&Ts), methyl decanoate (Es) with 4-terpineol (Te&Ts), and *β*-ocimene (Te&Ts) with 3-octanone (Ket). Different from CC at T1, linalool (14.8%; Te&Ts; No. **45** in Table 4) was the main volatile compound in LC at T1, followed by chloroform (13.3%; HoAlka; No. **5**) and linalyl isobutyrate (13.1%; Te&Ts; No. **47**), and then by caproic acid (4.9%; CA; No. **59**), an isomer of trans-linalool oxide (3.9%; Te&Ts; No. **36**), 1-hexanol (3.8%; Alc; No. **31**), isopropanol (3.7%; Alc; No. **2**), acetonitrile (3.5%; CyAlka; No. **4**), and 1-butanol (3.1%; Alc; No. **12**). The number of volatile constituents in LC did not change with ripening time, but their amount increased from 16,366.71 µg/kg cheese at T1 to 29,619.78 µg/kg cheese at T2 and decreased to 19,695.86 µg/kg cheese at T3. As for CC, the total amount of volatile compounds increased during ripening from 3337.08 µg/kg cheese at T1 to 10,392.16 µg/kg cheese at T2 and further to 14,743.17 µg/kg cheese at T3. The content of total volatile compounds in the Gouda-type cheese was 79.6% higher in LC than in CC at T1, with 64.9% at T2 and 25.1% at T3. As can be noticed in Figure 3, the concentration of volatile compounds either decreased with ripening time, varied in a ˄-pattern, or remained unchanged (cluster 1); in other cases, it increased (cluster 2).

**Table 4:** Volatile compound concentrations in control and lavender Gouda-type cheese at different ripening times (T1, T2, and T3).

Crt.No.	Volatile Compound	MSS (%)	Odor Descriptor	Reference	CC	Control Cheese			Lavender Cheese		
						µg/kg Cheese					
						T1	T2	T3	T1	T2	T3
1	Methyl acetate	96	Fruity, solvent, blackcurrant-like	[48]	Es	38.56 ± 4.199^bB^	67.26 ± 2.948^bB^	111.20 ± 26.935^aA^	53.50 ± 1.607^bA^	115.42 ± 25.312^aA^	61.89 ± 5.974^bB^
2	Isopropanol	97	Sharp musty	[49]	Alc	221.75 ± 46.558^cB^	722.34 ± 89.846^bB^	1155.17 ± 222.182^aA^	599.38 ± 75.037^bA^	1047.99 ± 120.237^aA^	590.10 ± 7.424^bB^
3	Methyl butyrate	96	Fruity, apple-like	[50]	Es	54.21 ± 11.983^bB^	114.20 ± 17.312^aB^	153.96 ± 31.418^aA^	180.06 ± 25.014^aA^	222.23 ± 34.620^aA^	170.39 ± 1.721^aA^
4	Acetonitrile	98	-	-	CyAlka	275.86 ± 51.660^cB^	1083.19 ± 169.607^bA^	1636.99 ± 279.534^aA^	575.97 ± 89.883^cA^	1495.49 ± 197.115^aA^	1022.22 ± 52.569^bB^
5	Chloroform	77	Ether-like	[49]	HoAlka	1068.81 ± 206.553^cB^	3527.63 ± 402.152^bA^	5590.37 ± 1139.680^aA^	2176.24 ± 339.353^bA^	4329.89 ± 565.593^aA^	4329.89 ± 565.593^aA^
6	Toluene	94	Nutty, bitter, almonds, paint; fruity	[51,52]	AH	23.03 ± 3.945^b^	51.99 ± 9.667^abA^	65.66 ± 18.515^aA^	n.d.	68.95 ± 22.883^aA^	41.11 ± 5.903^aA^
7	Ethyl butyrate (isomer)	95	Fruity, pineapple, acetone, caramel	[48]	Es	n.d.	n.d.	n.d.	39.08 ± 2.587^a^	50.16 ± 15.106^a^	28.71 ± 12.886^a^
8	Butyl acetate	94	Fruity	[49]	Es	15.34 ± 3.106^aB^	25.07 ± 3.967^aA^	23.29 ± 5.877^aA^	27.71 ± 2.459^aA^	22.75 ± 2.323^bA^	13.80 ± 0.133^cB^
9	Hexanal	95	Cut grass	[53]	Ald	16.51 ± 5.051^aA^	6.68 ± 2.494^bA^	n.d.	31.52 ± 20.601^aA^	6.86 ± 2.523^aA^	19.44 ± 0.720^a^
10	Isobutanol	91	Choking alcohol	[49]	Alc	6.31 ± 1.424^bB^	24.40 ± 3.487^aB^	28.14 ± 8.926^aA^	12.67 ± 3.009^cA^	34.31 ± 1.756^aA^	20.10 ± 3.050^bA^
11	Isoamyl acetate	97	Banana, chewing gum	[48]	Es	n.d.	n.d.	n.d.	n.d.	62.98 ± 10.744^a^	52.95 ± 4.624^a^
12	1-Butanol	97	Medicinal, fruity	[51]	Alc	201.21 ± 41.648^cB^	640.06 ± 83.138^bA^	950.89 ± 166.505^aA^	505.86 ± 71.458^aA^	638.14 ± 80.605^aA^	319.35 ± 28.604^bB^
13	*β*-Myrcene	93	Balsamic, spice	[54]	Te&Ts	n.d.	n.d.	n.d.	29.92 ± 9.055^b^	62.30 ± 6.066^a^	38.28 ± 2.828^b^
14	Pyridine	92	-	-	Pyr	12.38 ± 3.015^bA^	50.35 ± 9.850^aA^	63.41 ± 11.766^aA^	21.12 ± 6.264^aA^	21.45 ± 2.673^aB^	10.31 ± 1.399^bB^
15	2-Heptanone	88	Fruity	[52]	Ket	6.17 ± 1.257^cA^	13.62 ± 2.483^bB^	23.78 ± 1.680^aA^	10.71 ± 2.732^bA^	30.92 ± 3.245^aA^	27.73 ± 2.546^aA^
16	Heptanal	80	Sour milk/dairy	[52]	Ald	5.94 ± 1.317	n.d.	n.d.	n.d.	n.d.	n.d.
17	Methyl hexanoate	96	Fruity, ester-like	[50]	Es	141.11 ± 25.302^bB^	288.30 ± 40.965^aB^	321.42 ± 53.508^aA^	428.97 ± 58.989^aA^	496.81 ± 69.633^aA^	279.30 ± 29.445^bA^
18	1,8-Cineole	97	Floral, minty, fruity	[54]	Te&Ts	n.d.	n.d.	n.d.	370.96 ± 56.128^b^	678.05 ± 61.261^a^	356.96 ± 17.757^b^
19	Isoamyl alcohol	97	Banana	[55]	Alc	194.44 ± 43.626^bB^	750.88 ± 94.873^aB^	831.51 ± 172.253^aA^	433.41 ± 95.289^cA^	1284.59 ± 151.738^aA^	758.69 ± 54.400^bA^
20	Ethyl butyrate (isomer)	96	Pineapple-like	[49]	Es	8.62 ± 2.144^bB^	14.59 ± 2.407^bB^	29.48 ± 7.633^aA^	79.59 ± 8.303^aA^	74.89 ± 2.553^aA^	54.02 ± 9.024^bB^
21	trans-*β*-Ocimene	82	Mushroom-like	[56]	Te&Ts	21.33 ± 4.752^cA^	50.26 ± 4.803^bB^	66.13 ± 7.807^aB^	97.95 ± 13.079^bB^	158.27 ± 8.496^aA^	90.65 ± 9.332^bA^
22	*ɣ*-Terpinene	83	Unpleasant	[57]	Te&Ts	5.57 ± 0.261^bB^	13.84 ± 1.000^aB^	14.45 ± 1.618^aA^	17.75 ± 2.660^bA^	30.85 ± 2.920^aA^	16.42 ± 1.305^bA^
23	Styrene	87	Sweet smell	[49]	AH	15.76 ± 1.719^bB^	42.52 ± 7.501^abB^	66.04 ± 17.135^aA^	32.90 ± 3.271^bA^	61.74 ± 5.802^aA^	35.94 ± 4.774^bB^
24	3-Octanone *	89	Mushroom-like/buttery	[58]	Ket	n.d.	n.d.	n.d.	192.64 ± 49.231^b^	400.72 ± 37.208^a^	181.33 ± 18.574^b^
25	*β*-Ocimene	87	Sweet, herb	[54]	Te&Ts	18.66 ± 2.322^b^	34.83 ± 9.189^a^	42.49 ± 4.247^a^	n.d.	n.d.	n.d.
26	*o*-Cymene	96	Gasoline, citrus	[54,59]	Te&Ts	56.15 ± 11.023^bB^	153.78 ± 18.436^aA^	151.50 ± 28.686^aA^	94.46 ± 15.536^bA^	172.38 ± 21.329^aA^	86.03 ± 15.595^bB^
27	Isoamyl butyrate	90	Green, fruity	[60]	Es	n.d.	n.d.	n.d.	25.73 ± 5.723^b^	66.30 ± 16.486^a^	55.30 ± 5.393^a^
28	Acetoin	96	Sour milk	[51]	Ket	185.42 ± 44.259^cB^	484.17 ± 63.701^aB^	331.24 ± 59.406^bA^	439.72 ± 24.064^bA^	1026.19 ± 125.392^aA^	283.79 ± 6.800^bA^
29	3-Methylacetoin	87	-	-	Ket	6.87 ± 0.932^bB^	13.14 ± 0.796^aA^	8.24 ± 1.433^bB^	18.18 ± 2.239^aA^	17.12 ± 4.537^aA^	17.12 ± 4.537^aA^
30	1-Butoxy-2-propanol	94	-	-	Alc	7.00 ± 1.595^bB^	33.38 ± 5.554^aB^	25.65 ± 4.623^aA^	33.53 ± 4.275^bA^	58.20 ± 7.734^aA^	28.39 ± 3.179^bA^
31	1-Hexanol	96	Sweet, green	[49]	Alc	28.91 ± 5.329^aB^	62.36 ± 46.499^aB^	95.27 ± 14.049^aB^	623.90 ± 309.213^aA^	552.70 ± 159.944^aA^	528.37 ± 238.769^aA^
32	1-Octenyl acetate	95	-	-	Es	n.d.	n.d.	n.d.	426.19 ± 57.704^b^	775.69 ± 80.136^a^	402.63 ± 25.727^b^
33	3-Octanol	84	Mushroom, cheese	[54]	Alc	n.d.	n.d.	n.d.	52.82 ± 5.691^b^	89.54 ± 9.653^a^	67.24 ± 10.446^b^
34	Hexyl butyrate	93	Fruity, green	[61]	Es	n.d.	n.d.	n.d.	17.50 ± 2.985^b^	34.34 ± 1.539^a^	19.27 ± 3.282^b^
35	Acetic acid	95	Vinegar, sour, sharp, peppery, green	[51]	CA	92.92 ± 21.149^bB^	445.64 ± 79.887^aA^	605.66 ± 92.910^aA^	226.10 ± 45.177^cA^	562.83 ± 68.060^aA^	363.00 ± 28.421^bB^
36	trans-Linalool oxide (isomer)	95	-	-	Te&Ts	n.d.	n.d.	n.d.	638.94 ± 108.438^b^	1247.76 ± 186.259^a^	633.61 ± 60.692^b^
37	1-Octen-3-ol	97	Mushroom, metallic	[54]	Alc	5.90 ± 1.212^bB^	10.13 ± 0.379^abB^	11.35 ± 2.807^aB^	84.88 ± 9.979^bA^	160.27 ± 25.469^aA^	82.59 ± 2.777^bA^
38	trans-2-Pinanol	86	-	-	Te&Ts	n.d.	n.d.	n.d.	17.53 ± 1.531^b^	30.69 ± 3.142^a^	19.49 ± 3.055^b^
39	trans-Linalool oxide (isomer)	97	-	-	Te&Ts	n.d.	n.d.	n.d.	327.46 ± 49.616^b^	558.54 ± 25.266^a^	305.77 ± 29.301^b^
40	Linalyl acetate	72	Flower, fruit, lavender	[54]	Te&Ts	n.d.	n.d.	n.d.	6.97 ± 0.964^b^	10.38 ± 0.517^a^	6.33 ± 0.437^b^
41	2-Ethyl-1-hexanol	90	Mild, oily, sweet, slightly floral odor reminiscent of rose	[49]	Alc	n.d.	n.d.	n.d.	11.11 ± 1.098^a^	11.17 ± 1.211^a^	10.57 ± 1.624^a^
42	Camphor	93	Camphor	[54]	Te&Ts	n.d.	n.d.	n.d.	61.08 ± 7.291^b^	101.92 ± 6.717^a^	54.25 ± 4.126^b^
43	Benzaldehyde	97	Almonds, sugar, burnt	[51]	Ald	49.32 ± 11.585^bB^	116.58 ± 14.396^aB^	115.88 ± 24.301^aB^	211.96 ± 12.459^aA^	271.88 ± 66.353^aA^	266.70 ± 52.937^aA^
44	2,3-Butanediol (optical isomer)	95	Fruity, onions	[51]	Alc	28.42 ± 9.483^cA^	77.80 ± 11.120^bB^	145.40 ± 24.605^aA^	39.73 ± 5.638^bA^	156.05 ± 25.139^aA^	119.33 ± 23.194^aA^
45	Linalool	86	Flower, lavender	[54,62]	Te&Ts	9.49 ± 1.481^abB^	8.77 ± 0.928^bB^	15.56 ± 3.930^aB^	2416.24 ± 313.723^bA^	4130.24 ± 295.527^aA^	2371.14 ± 188.355^bA^
46	Isobutyric acid	94	Cheesy, butter, rancid	[51]	CA	12.31 ± 1.076^c^	81.03 ± 16.303^b^	142.94 ± 26.383^a^	n.d.	n.d.	n.d.
47	Linalyl isobutyrate **	87	Cheesy, butter, rancid	[51]	Te&Ts	n.d.	n.d.	n.d.	2143.98 ± 264.564^b^	3817.29 ± 361.877^a^	2297.54 ± 183.074^b^
48	2,3-Butanediol (meso isomer)	97	Fruity, onions	[51]	Alc	81.21 ± 31.384^cA^	175.49 ± 32.577^bB^	307.94 ± 34.889^aA^	98.55 ± 13.951^bA^	478.83 ± 157.897^aA^	215.29 ± 86.648^bA^
49	4-Terpineol ***	86	Green, liquorice, moldy	[48]	Te&Ts	n.d.	n.d.	n.d.	237.05 ± 30.071^b^	373.97 ± 35.340^a^	258.37 ± 31.058^b^
50	Methyl decanoate	89	-	-	Es	13.02 ± 1.438^c^	30.64 ± 4.524^b^	40.91 ± 4.701^a^	n.d.	n.d.	n.d.
51	Lavandulyl acetate	93	Floral odor reminiscent of lavender	[63]	Te&Ts	n.d.	n.d.	n.d.	211.16 ± 29.910^b^	355.30 ± 35.686^a^	216.28 ± 19.026^b^
52	Butyric acid	93	Parmesan cheese, vomit, butanoic acid	[48]	CA	81.43 ± 21.084^cB^	212.27 ± 56.676^bB^	389.59 ± 64.874^aA^	469.11 ± 26.717^aA^	643.62 ± 144.232^aA^	506.19 ± 81.424^aA^
53	Phenylacetaldehyde	94	Floral, rosy, sweet, hyacinth-like	[62]	Ald	n.d.	n.d.	n.d.	20.02 ± 1.922^a^	23.38 ± 1.934^a^	12.98 ± 3.641^b^
54	Isovaleric acid	94	Sweet, rancid, rotten, cheesy, Swiss cheese	[51]	CA	64.68 ± 6.667^cB^	373.42 ± 17.242^bB^	537.57 ± 30.138^aB^	207.08 ± 13.678^cA^	553.87 ± 37.967^bA^	702.86 ± 33.921^aA^
55	*α*-Terpineol	83	Oil, anise, mint	[54]	Te&Ts	n.d.	n.d.	n.d.	20.99 ± 2.956^b^	31.51 ± 2.595^a^	18.49 ± 3.808^b^
56	Borneol	95	Camphor	[54]	Te&Ts	n.d.	n.d.	n.d.	155.76 ± 21.932^b^	250.67 ± 27.919^a^	145.19 ± 32.109^b^
57	cis-Geranyl acetate	86	Rose, fruity, flower	[54]	Te&Ts	n.d.	n.d.	n.d.	34.31 ± 3.226^b^	56.58 ± 6.103^a^	34.09 ± 6.532^b^
58	Linalool oxide (isomer)	77	Floral, honey-like	[61]	Te&Ts	n.d.	n.d.	n.d.	10.48 ± 2.264^b^	15.63 ± 1.521^a^	9.73 ± 2.250^b^
59	Caproic acid	93	Bad breath, popcorn, goaty	[51]	CA	185.93 ± 46.042^bB^	403.05 ± 90.032^aB^	451.28 ± 36.396^aB^	807.79 ± 45.073^bA^	1133.32 ± 206.390^aA^	722.20 ± 16.136^bA^
60	2,6-dimethyl-3,7-octadien-2,6-diol	83	-	-	Alc	n.d.	n.d.	n.d.	37.95 ± 6.169^b^	65.88 ± 5.583^a^	43.95 ± 2.542^b^
61	Caryophyllene oxide	87	Terpene notes, weak woody–spicy	[64]	Te&Ts	n.d.	n.d.	n.d.	26.76 ± 3.983^b^	47.81 ± 1.866^a^	31.78 ± 4.108^b^
62	Caprylic acid	97	Sweaty, rancid	[51]	CA	76.51 ± 7.885^bB^	188.50 ± 28.549^aB^	192.79 ± 32.975^aA^	193.80 ± 21.998^bA^	342.19 ± 29.252^aA^	240.40 ± 41.430^bA^
					**TOTAL, of which:**	**3337.08**	**10,392.16**	**14,743.17**	**16,366.71**	**29,619.78**	**19,695.86**
					Alc	775.14	2496.84	3551.32	2533.80	4577.67	2783.98
					Ald	71.77	123.26	115.88	263.50	302.12	299.12
					AH	38.79	94.52	131.71	32.90	130.69	77.05
					CA	513.79	1703.91	2319.84	1903.87	3235.83	2534.65
					CyAlka	275.86	1083.19	1636.99	575.97	1495.49	1022.22
					Es	270.86	540.06	680.26	1278.34	1921.57	1138.26
					HoAlka	1068.81	3527.63	5590.37	2176.24	4329.89	4329.89
					Ket	198.46	510.93	363.26	661.25	1474.94	509.98
					Pyr	12.38	50.35	63.41	21.12	21.45	10.31
					Te&Ts	111.21	261.48	290.13	6919.72	12130.13	6990.40

* 3-octanone was identified along with *β*-ocimene in LC; ** linalyl isobutyrate was identified along with isobutyric acid in LC; *** 4-terpineol was identified along with methyl decanoate in LC; MSS—mass spectra similarity; T1—10 days of ripening; T2—20 days of ripening; T3—30 days of ripening; CC—chemical class; Es—esters; Alc—alcohols; CyAlka—cyanoalkanes; HoAlka—haloalkanes; AH—aromatic hydrocarbons; Ald—aldehydes; Te&Ts—terpene and terpenoids; Pyr—pyridines; Ket—ketones; CA—carboxylic acids; n.d.—not detected. Data are expressed as mean ± standard deviation values of all measurements. Different lowercase letters in the same row indicate significant differences between ripening times (*p* < 0.05, Tukey’s test), and different uppercase letters show significant differences between cheeses (*p* < 0.05).

### 3.5. Sensory Properties of Gouda-Type Cheese and Consumers’ Acceptance and Purchase Intention

**Figure 4:**
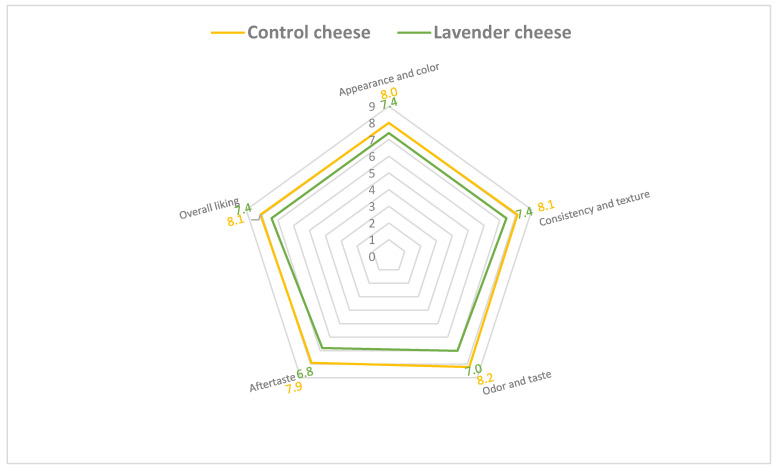
Hedonic scores for sensory attributes of control and lavender Gouda-type cheese. Data are expressed as mean values of all responses.

**Figure 5:**
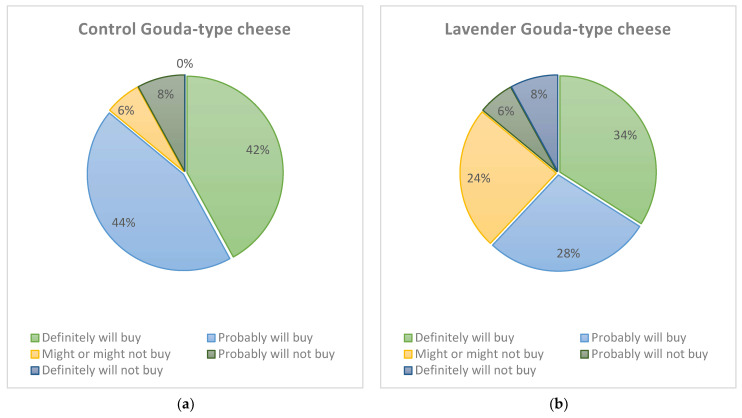
Response rates (%) for purchase intention of (**a**) control Gouda-type cheese; (**b**) lavender Gouda-type cheese.

Consumers’ perception of LC compared to CC was evaluated based on hedonic scores of their sensory attributes (Figure 4). The use of lavender flower powder as a flavoring ingredient in Gouda-type cheese-making has significantly affected its sensory properties by reducing its rating for appearance and color (0.6 points), consistency and texture (0.7 points), odor and taste (1.2 points), aftertaste (1.1 points), and overall liking (0.7 points). The overall score for LC was 7.2, and that for CC was 8.1. However, the calculation resulted in an acceptance rate of 80.2% for LC and 89.5% for CC. Furthermore, when asked about their willingness to buy the Gouda-type cheese, 34% of respondents answered that they “definitely will buy” LC, and 42% responded in this way for CC (see Figure 5). As regards the undecided subjects, 24% answered “might or might not buy” and 8% “probably will buy” the LC, which shows greater indecision about the flavored cheese. This outcome highlights the lack of familiarity but a curiosity towards this new product. For LC, 8% of survey participants responded with “definitely will not buy”. Overall, these results show that there would be customers for LC if this were available on the market.

## 4. Conclusions

The manufacturing process proposed in this study resulted in a Gouda-type cheese formulation with a lavender aroma. Using lavender flower powder as a flavoring ingredient at a concentration of 0.5 g/L matured milk in Gouda-type cheese manufacturing conferred upon the cheese a terpenic volatile profile and stimulated the growth of lactic acid bacteria from the starter culture. During ripening, a concentration of volatile compounds and lactic acid bacteria, both in the control and lavender cheese, and an improvement in their nutritional and textural properties was noticed. However, it should be underlined that the flavoring ingredient did not significantly impact the Gouda-type cheese’s gross composition and textural properties. Regarding the sensory perception of the lavender cheese, it should be noted that its overall score was slightly lower but very close to that received by the unflavored cheese, definitively more familiar. Although the acceptance rate of the lavender-flavored cheese was high, consumers were less willing to buy it, being a gourmet product. However, with effective communication of this new product, perhaps accompanied by a food pairing study, the lavender-flavored Gouda-type cheese could be a successful novelty in the worldwide dairy market.

## Figures and Tables

**Table 1:** Proximate chemical composition, sodium chloride content, pH, titratable acidity, and energy value of control and lavender Gouda-type cheese at different ripening times (T1, T2, and T3).

Parameter/Energy Value	Control Cheese			Lavender Cheese		
	T1	T2	T3	T1	T2	T3
Moisture (%)	36.84 ± 0.270^aA^	35.80 ± 0.060^abA^	34.97 ± 0.064^bA^	37.87 ± 0.282^aA^	36.57 ± 0.630^abA^	35.48 ± 0.165^bA^
Protein (%)	21.13 ± 0.191^cA^	22.11 ± 0.014^bA^	23.17 ± 0.212^aB^	21.55 ± 0.078^cA^	22.39 ± 0.134^bA^	24.90 ± 0.113^aA^
Fat (%)	28.25 ± 0.354^cA^	29.50 ± 0.0^bA^	30.75 ± 0.354^aA^	29.25 ± 0.354^aA^	29.25 ± 0.354^aA^	29.75 ± 0.354^aA^
Fat in dry matter (%)	44.73 ± 0.057^cB^	45.95 ± 0.042^bA^	47.29 ± 0.495^aA^	47.08 ± 0.354^aA^	46.11 ± 0.099^aA^	46.11 ± 0.431^aA^
Ash (%)	4.28 ± 0.014^bA^	4.49 ± 0.156^bA^	5.45 ± 0.014^aA^	4.25 ± 0.007^bA^	4.31 ± 0.035^bA^	5.06 ± 0.028^aB^
Total carbohydrate (%)	9.51 ± 0.700^aA^	8.10 ± 0.205^aA^	5.66 ± 0.488^bA^	7.09 ± 0.0^aB^	7.50 ± 0.177^aA^	4.82 ± 0.106^bA^
Sodium chloride (%)	2.37 ± 0.028^cA^	2.60 ± 0.021^bB^	2.71 ± 0.007^aB^	2.26 ± 0.028^cA^	2.91 ± 0.028^bA^	3.28 ± 0.071^aA^
pH	4.87 ± 0.014^aB^	4.73 ± 0.007^bA^	4.60 ± 0.0^cA^	4.99 ± 0.007^aA^	4.83 ± 0.092^abA^	4.65 ± 0.042^bA^
Titratable acidity (°T)	85.2 ± 0.566^cA^	90.6 ± 0.849^bA^	95.0 ± 0.283^aA^	79.8 ± 0.283^cB^	83.8 ± 0.283^bB^	92.0 ± 0.0^aB^
Energy value (kcal/100 g)	377 ± 4.950^bA^	387 ± 0.707^abA^	393 ± 2.121^aA^	378 ± 2.828^aA^	383 ± 4.243^aA^	387 ± 2.121^aA^

T1—10 days of ripening; T2—20 days of ripening; T3—30 days of ripening. Data are expressed as mean ± standard deviation values of all measurements. Different lowercase letters in the same row indicate significant differences between ripening times (*p* < 0.05, Tukey’s test), and different uppercase letters show significant differences between cheeses (*p* < 0.05).

**Table 2:** Texture attribute values for control and lavender Gouda-type cheese at different ripening times (T1, T2, and T3).

Texture Attribute	Control Cheese			Lavender Cheese		
	T1	T2	T3	T1	T2	T3
Hardness (N)	35.43 ± 2.456^bA^	36.52 ± 5.547^bA^	53.07 ± 10.105^aA^	24.72 ± 3.534^bB^	40.56 ± 10.605^aA^	41.51 ± 9.777^aA^
Cohesiveness	0.46 ± 0.066^aA^	0.37 ± 0.047^bA^	0.24 ± 0.028^cA^	0.44 ± 0.061^aA^	0.29 ± 0.036^bB^	0.24 ± 0.030^bA^
Springiness index	0.83 ± 0.026^aA^	0.79 ± 0.061^aA^	0.54 ± 0.076^bA^	0.82 ± 0.088^aA^	0.80 ± 0.048^aA^	0.68 ± 0.019^bA^
Gumminess (N)	16.09 ± 2.530^aA^	19.90 ± 6.244^aA^	8.94 ± 2.153^bA^	10.72 ± 0.987^aB^	11.72 ± 2.580^aB^	10.17 ± 3.366^aA^
Chewiness index (N)	13.35 ± 2.229^aA^	15.71 ± 5.299^aA^	4.90 ± 1.587^bA^	8.57 ± 1.121^aB^	9.43 ± 2.914^aB^	6.96 ± 1.060^bA^

T1—10 days of ripening; T2—20 days of ripening; T3—30 days of ripening. Data are expressed as mean ± standard deviation values of all measurements. Different lowercase letters in the same row indicate significant differences between ripening times (*p* < 0.05, Tukey’s test), and different uppercase letters show significant differences between cheeses (*p* < 0.05).

**Table 3:** Counts of lactobacilli, streptococci, and total lactic acid bacteria in control and lavender Gouda-type cheese at different ripening times (T1, T2, and T3).

Parameter	Control Cheese			Lavender Cheese		
	T1	T2	T3	T1	T2	T3
Lactobacilli count	9.5 ± 0.007^cB^	9.7 ± 0.009^bB^	9.8 ± 0.004^aB^	9.7 ± 0.005^cA^	9.8 ± 0.004^bA^	9.9 ± 0.006^aA^
Streptococci count	8.7 ± 0.011^cB^	9.2 ± 0.011^bB^	9.3 ± 0.016^aB^	9.0 ± 0.024^cA^	9.5 ± 0.012^bA^	9.6 ± 0.013^aA^
Total lactic acid bacteria count	18.2 ± 0.004^cB^	18.9 ± 0.002^bB^	19.1 ± 0.020^aB^	18.7 ± 0.019^cA^	19.3 ± 0.008^bA^	19.5 ± 0.020^aA^

T1—10 days of ripening; T2—20 days of ripening; T3—30 days of ripening. Data are expressed as mean ± standard deviation values of all enumerations. Different lowercase letters in the same row indicate significant differences between ripening times (*p* < 0.05, Tukey’s test), and different uppercase letters show significant differences between cheeses (*p* < 0.05).

## Data Availability

All data generated or analyzed during this study are included in this published article.

## References

[1] Codex Alimentarius Commission Standard for Gouda CXS 266-1966. https://www.fao.org/fao-who-codexalimentarius/sh-proxy/en/?lnk=1&url=https%253A%252F%252Fworkspace.fao.org%252Fsites%252Fcodex%252FStandards%252FCXS%2B266-1966%252FCXS_266e.pdf.

[2] Chen C., Tian T., Yu H., Yuan H., Wang B., Xu Z., Tian H. (2022). Characterisation of the key volatile compounds of commercial Gouda cheeses and their contribution to aromas according to Chinese consumers’ preferences. Food Chem. X.

[3] Choi H.Y., Yang C.J., Choi K.S., Bae I. (2015). Characteristics of Gouda cheese supplemented with fruit liquors. J. Anim. Sci. Technol..

[4] Semeniuc C.A., Zăpârţan L., Stan L., Pop C.R., Borş M.D., Rotar A.M. (2015). Physicochemical and sensory properties of whey cheese with pine nuts. Bull. UASVM Food Sci. Technol..

[5] Zhang X.Y., Guo H.Y., Zhao L., Sun W.F., Zeng S.S., Lu X.M., Cao X., Ren F.Z. (2011). Sensory profile and Beijing youth preference of seven cheese varieties. Food Qual. Prefer..

[6] Semeniuc C.A., Mandrioli M., Socaci B.S., Socaciu M.-I., Fogarasi M., Podar A.S., Michiu D., Jimborean A.M., Mureşan V., Ionescu S.R. (2022). Changes in lipid composition and oxidative status during ripening of Gouda-type cheese as influenced by addition of lavender flower powder. Int. Dairy J..

[7] Taherkhani P., Noori N., Akhondzadeh Basti A., Gandomi H., Alimohammadi M. (2015). Antimicrobial effects of Kermanian black cumin (*Bunium persicum* Boiss.) essential oil in Gouda cheese matrix. J. Med. Plants.

[8] Kim Y.K., Nam M.S., Bae H.C. (2017). Characteristics of Gouda cheese supplemented with chili pepper extract microcapsules. Korean J. Food Sci. Anim. Resour..

[9] Düsterhöft E.M., Engels W., Huppertz T., Papademas P., Bintsis T. (2018). Dutch-type cheeses. Global Cheesemaking Technology: Cheese Quality and Characteristics.

[10] Majdik A. Experimental Studies on Changes Made to Pre Press for Gouda Cheese with Spices. Proceedings of the International Multidisciplinary Scientific GeoConference: SGEM.

[11] Agboola S.O., Radovanovic-Tesic M. (2002). Influence of Australian native herbs on the maturation of vacuum-packed cheese. LWT-Food Sci. Technol..

[12] Park W., Yoo J., Oh S., Ham J.S., Jeong S.G., Kim Y. (2019). Microbiological characteristics of Gouda cheese manufactured with pasteurized and raw milk during ripening using next generation sequencing. Food Sci. Anim. Resour..

[13] Open Universiteit, Thames Polytechnic, Open Universiteit, Thames Polytechnic (1991). Starter cultures for cheese production. CIP: Biotechnological Innovations in Food Processing.

[14] (2004). Cheese and Processed Cheese—Determination of the Total Solids Content (Reference Method).

[15] (2014). Milk and Milk Products—Determination of Nitrogen Content—Part 1: Kjeldahl Principle and Crude Protein Calculation.

[16] (2008). Cheese—Determination of Fat Content—Van Gulik Method.

[17] Nagy M., Semeniuc C.A., Socaci S.A., Pop C.R., Rotar A.M., Sălăgean C.D., Tofană M. (2017). Utilization of brewer’s spent grain and mushrooms in fortification of smoked sausages. Food Sci. Technol..

[18] (2006). Cheese and Processed Cheese Product—Determination of Chloride Conten—Potentiometric Titration Method.

[19] (1985). Milk and Milk Products—Determination of Acidity.

[20] Ong L., Dagastine R.R., Kentish S.E., Gras S.L. (2012). The effect of pH at renneting on the microstructure, composition and texture of Cheddar cheese. Food Res. Int..

[21] Socaciu M.I., Fogarasi M., Simon E.L., Semeniuc C.A., Socaci S.A., Podar A.S., Vodnar D.C. (2021). Effects of whey protein isolate-based film incorporated with tarragon essential oil on the quality and shelf-life of refrigerated brook trout. Foods.

[22] (2003). Yogurt—Enumeration of Characteristic Microorganisms—Colony-Count Technique at 37 °C.

[23] Cozzolino R., Martignetti A., De Giulio B., Malorni L., Addeo F., Picariello G. (2021). SPME GC-MS monitoring of volatile organic compounds to assess typicity of Pecorino di Carmasciano ewe-milk cheese. Int. J. Dairy Technol..

[24] Lignou S., Oloyede O.O. (2021). Consumer acceptability and sensory profile of sustainable paper-based packaging. Foods.

[25] dos Reis Santos J., Gomes Hafemann S.P., Giani Pieretti G., Antigo J.L., Soares dos Santos Pozza M., da Silva Scapim M.R., Scaramal Madrona G. (2014). Sensorial, microbiological, and physico-chemical analysis of Minas frescal cheese with oregano essential oil (*Origanum vulgare*) addition. Int. J. Food Sci. Nutr. Eng..

[26] Wang Y., Wu J., Lv M., Shao Z., Hungwe M., Wang J., Bai X., Xie J., Wang Y., Geng W. (2021). Metabolism characteristics of lactic acid bacteria and the expanding applications in food industry. Front. Bioeng. Biotechnol..

[27] Mureşan C.C., Marc Vlaic R.A., Semeniuc C.A., Socaci A.S., Fărcaş A., Francisc D., Pop C.R., Rotar A., Dodan A., Mureşan V. (2021). Changes in physicochemical and microbiological properties, fatty acid and volatile compound profiles of Apuseni cheese during ripening. Foods.

[28] Kanawjia S.K., Rajesh P., Sabikhi L., Singh S. (1995). Flavour, chemical and textural profile changes in accelerated ripened Gouda cheese. LWT-Food Sci. Technol..

[29] Ivanov G., Bogdanova A., Zsivanovits G. (2018). Effect of ripening temperature on the texture of cow milk Kashkaval cheese. Prog. Agri. Eng. Sci..

[30] Zheng Y., Liu Z., Mo B. (2016). Texture profile analysis of sliced cheese in relation to chemical composition and storage temperature. J. Chem..

[31] Pinho O., Mendes E., Alves M.M., Ferreira I.M.P.L.V.O. (2004). Chemical, physical, and sensorial characteristics of “Terrincho” ewe cheese: Changes during ripening and intravarietal comparison. J. Dairy Sci..

[32] Librán C.M., Moro A., Zalacain A., Molina A., Carmona M., Berruga M.I. (2013). Potential application of aromatic plant extracts to prevent cheese blowing. World J. Microbiol. Biotechnol..

[33] Öztürk H.İ., Aydın S., Akın N. (2017). Effect of lavender powder on microbial, physicochemical, sensory and functional properties of yoghurt. Int. J. Second. Metab..

[34] Kınık O., Kesenkaş H., Ergönül P.G., Akan E. (2017). The effect of using pro and prebiotics on the aromatic compounds, textural and sensorial properties of symbiotic goat cheese. Mljekarstvo.

[35] Hayaloglu A.A., Karabulut I. (2013). SPME/GC-MS characterization and comparison of volatiles of eleven varieties of Turkish cheeses. Int. J. Food Prop..

[36] Gómez-Ruiz J.Á., Ballesteros C., González Viñas M.Á., Cabezas L., Martínez-Castro I. (2002). Relationships between volatile compounds and odour in Manchego cheese: Comparison between artisanal and industrial cheeses at different ripening times. Lait.

[37] Resch P., Guthy K. (1999). Chloroform in milk and dairy products. Part A: Analysis of chloroform using static headspace gaschromatography. Dtsch Leb. Rundsch.

[38] Van Leuven I., Van Caelenberg T., Dirinck P. (2008). Aroma characterisation of Gouda-type cheeses. Int. Dairy J..

[39] Van Hoorde K., Van Leuven I., Dirinck P., Heyndrickx M., Coudijzer K., Vandamme P., Huys G. (2010). Selection, application and monitoring of *Lactobacillus paracasei* strains as adjunct cultures in the production of Gouda-type cheeses. Int. J. Food Microbiol..

[40] Ordiales E., Martín A., Benito M.J., Hernández A., Ruiz-Moyano S., de Guía Córdoba M. (2013). Role of the microbial population on the flavor of the soft-bodied cheese Torta del Casar. J. Dairy Sci..

[41] Aminifar M., Hamedi M., Emam-Djomeh Z., Mehdinia A. (2014). Investigation on proteolysis and formation of volatile compounds of Lighvan cheese during ripening. J. Food Sci. Technol..

[42] Jung H.J., Ganesan P., Lee S.J., Kwak H.S. (2013). Comparative study of flavor in cholesterol-removed Gouda cheese and Gouda cheese during ripening. J. Dairy Sci..

[43] Ruyssen T., Janssens M., Van Gasse B., Van Laere D., Van der Eecken N., De Meerleer M., Vermeiren L., Van Hoorde K., Martins J.C., Uyttendaele M. (2013). Characterisation of Gouda cheeses based on sensory, analytical and high-field 1H nuclear magnetic resonance spectroscopy determinations: Effect of adjunct cultures and brine composition on sodium-reduced Gouda cheese. Int. Dairy J..

[44] Shiota M., Iwasawa A., Suzuki-Iwashima A., Iida F. (2015). Effects of flavor and texture on the sensory perception of Gouda-type cheese varieties during ripening using multivariate analysis. J. Food Sci..

[45] Jo Y., Benoist D.M., Ameerally A., Drake M.A. (2017). Sensory and chemical properties of Gouda cheese. J. Dairy Sci..

[46] Sýkora M., Vítová E., Jeleń H.H. (2020). Application of vacuum solid-phase microextraction for the analysis of semi-hard cheese volatiles. Eur. Food Res. Technol..

[47] Semeniuc C.A., Mandrioli M., Rodriguez-Estrada M.T., Muste S., Lercker G. (2016). Thiobarbituric acid reactive substances in flavored phytosterol-enriched drinking yogurts during storage: Formation and matrix interferences. Eur. Food Res. Technol..

[48] Varming C., Petersen M.A., Poll L. (2004). Comparison of isolation methods for the determination of important aroma compounds in black currant (*Ribes nigrum* L.) juice, using nasal impact frequency profiling. J. Agric. Food Chem..

[49] PubChem. https://pubchem.ncbi.nlm.nih.gov/.

[50] Zheng L.-Y., Sun G.-M., Liu Y.-G., Lv L.-L., Yang W.-X., Zhao W.-F., Wei C.-B. (2012). Aroma volatile compounds from two fresh pineapple varieties in China. Int. J. Mol. Sci..

[51] Lacroix N., St-Gelais D., Champagne C.P., Fortin J., Vuillemard J.-C. (2010). Characterization of aromatic properties of old-style cheese starters. J. Dairy Sci..

[52] O’Riordan P.J., Delahunty C.M. (2001). Comparison of volatile compounds released during the consumption of Cheddar cheese with compounds extracted by vacuum distillation using gas chromatography–olfactometry. Flavour. Fragr. J..

[53] Karagul Yuceer Y., Tuncel B., Guneser O., Engin B., Isleten M., Yasar K., Mendes M. (2009). Characterization of aroma-active compounds, sensory properties, and proteolysis in Ezine cheese. J. Dairy Sci..

[54] Xiao Z., Li Q., Niu Y., Zhou X., Liu J., Xu Y., Xu Z. (2017). Odor-active compounds of different lavender essential oils and their correlation with sensory attributes. Ind. Crop. Prod..

[55] Gómez-Míguez M.J., Cacho J.F., Ferreira V., Vicario I.M., Heredia F.J. (2007). Volatile components of Zalema white wines. Food Chem..

[56] Song H.S., Sawamura M., Ito T., Ido A., Ukeda H. (2000). Quantitative determination and characteristic flavour of daidai (*Citrus aurantium* L. var. *cyathifera* Y. Tanaka) peel oil. Flavour. Fragr. J..

[57] Senoussi A., Rapisarda T., Schadt I., Chenchouni H., Saoudi Z., Senoussi S., Zidoune O.A., Zidoune M.N., Carpino S. (2022). Formation and dynamics of aroma compounds during manufacturing-ripening of Bouhezza goat cheese. Int. Dairy J..

[58] Cho I.H., Namgung H.-J., Choi H.-K., Kim Y.-S. (2008). Volatiles and key odorants in the pileus and stipe of pine-mushroom (*Tricholoma matsutake* Sing.). Food Chem..

[59] Semeniuc C.A., Rotar A., Stan L., Pop C.R., Socaci S., Mireşan V., Muste S. (2016). Characterization of pine bud syrup and its effect on physicochemical and sensory properties of kefir. CyTA-J. Food.

[60] Tsouli Sarhir S., Amanpour A., Bouseta A., Selli S. (2022). Potent odorants and sensory characteristics of the soft white cheese “Jben”: Effect of salt content. Flavour. Fragr. J..

[61] Guo X., Ho C.-T., Wan X., Zhu H., Liu Q., Wen Z. (2021). Changes of volatile compounds and odor profiles in Wuyi rock tea during processing. Food Chem..

[62] Zhu M., Sun J., Zhao H., Wu F., Xue X., Wu L., Cao W. (2022). Volatile compounds of five types of unifloral honey in Northwest China: Correlation with aroma and floral origin based on HS-SPME/GC–MS combined with chemometrics. Food Chem..

[63] Guo X., Wang P. (2020). Aroma characteristics of lavender extract and essential oil from *Lavandula angustifolia* Mill. Molecules.

[64] Bail S., Buchbauer G., Schmidt E., Wanner J., Slavchev A., Stoyanova A., Denkova Z., Geissler M., Jirovetz L. (2008). GC-MS-analysis, antimicrobial activities and olfactory evaluation of essential davana (*Artemisia pallens* Wall. ex DC) oil from India. Nat. Prod. Commun..

